# A scoping review of artificial intelligence as a medical device for ophthalmic image analysis in Europe, Australia and America

**DOI:** 10.1038/s41746-025-01726-8

**Published:** 2025-05-29

**Authors:** Ariel Yuhan Ong, Priyal Taribagil, Mertcan Sevgi, Aditya U. Kale, Eliot R. Dow, Trystan Macdonald, Ashley Kras, Gregory Maniatopoulos, Xiaoxuan Liu, Pearse A. Keane, Alastair K. Denniston, Henry David Jeffry Hogg

**Affiliations:** 1 Moorfields Eye Hospital NHS Foundation Trust, London, UK; 2 Institute of Ophthalmology, University College London, London, UK; 3 NIHR Moorfields Biomedical Research Centre, London, UK; 4 Oxford Eye Hospital, Oxford University Hospitals NHS Foundation Trust, Oxford, UK; 5 University Hospitals Birmingham NHS Foundation Trust, Birmingham, UK; 6 College of Medicine and Health, University of Birmingham, Birmingham, UK; 7 Retinal Consultants Medical Group, Sacramento, CA USA; 8 Sydney Eye Hospital, Sydney, NSW Australia; 9 University of Leicester, Leicester, UK; 10 NIHR Birmingham Biomedical Research Centre, Birmingham, UK; 11 Birmingham Health Partners Centre for Regulatory Science and Innovation, Birmingham, UK

**Keywords:** Health care, Diagnosis

## Abstract

This scoping review aims to identify regulator-approved ophthalmic image analysis artificial intelligence as a medical device (AIaMD) in three jurisdictions, examine their characteristics and regulatory approvals, and evaluate the available evidence underpinning them, as a step towards identifying best practice and areas for improvement. 36 AIaMDs from 28 manufacturers were identified – 97% (35/36) approved in the EU, 22% (8/36) in Australia, and 8% (3/36) in the USA. Most targeted diabetic retinopathy detection. 19% (7/36) did not have published evidence describing performance. For the remainder, 131 clinical evaluation studies (range 1-22/AIaMD) describing 192 datasets/cohorts were identified. Demographics were poorly reported (age recorded in 52%, sex 51%, ethnicity 21%). On a study-level, few included head-to-head comparisons against other AIaMDs (8%,10/131) or humans (22%, 29/131), and 37% (49/131) were conducted independently of the manufacturer. Only 11 studies (8%) were interventional. There is scope for expanding AIaMD applications to other ophthalmic imaging modalities, conditions, and use cases. Facilitating greater transparency from manufacturers, better dataset reporting, validation across diverse populations, and high-quality interventional studies with implementation-focused outcomes are key steps towards building user confidence and supporting clinical integration.

## Introduction

Artificial intelligence (AI) represents a rapidly evolving frontier in healthcare which offers transformative potential across multiple specialties. In ophthalmology, AI can help enhance diagnostic accuracy, provide insights into systemic diseases, streamline clinical and research workflows, optimise treatment, with the ultimate goal of improving patient outcomes^1^. It can potentially help in addressing challenges such as variability in subjective human interpretation, the increasing volume of complex imaging data, and the global shortage of ophthalmic specialists. This shortage has resulted in a capacity-demand imbalance that risks irreversible sight loss from treatment delays^2–4^, affecting quality of life for patients and carers^5–7^, and posing a significant economic burden to individuals, healthcare services, and society^8,9^.

However, while AI promises significant advancements, it may also introduce new complexities in the evaluation of safety, effectiveness, equity and bias, which are essential to ensure they achieve their intended purpose for their target population^10^. At present, the evidence requirements to support the regulatory approval of artificial intelligence as a medical device (AIaMD) are less transparent compared to more established interventions such as drugs, which follow well-established and rigorous pathways for evaluation prior to reaching the market. Evidence standards for AIaMDs are informed by regulations which are designed to be broadly applicable across all medical devices and clinical contexts. This therefore requires some level of abstraction, which allows for varied interpretations when applied to specific AIaMDs and use cases. This raises questions about what constitutes a ‘sufficient’ level of evidence for regulatory approval or real-world deployment, particularly for AIaMDs intended to support or replace clinicians in their decision-making processes^11,12^.

Given this context, understanding the level and variation of evidence underpinning AIaMDs which have received regulatory approval for clinical use may help to identify best practices and opportunities for improvement in AIaMD evidence generation and appraisal. This would support the use of AIaMDs that are safe, effective, and beneficial for the populations they aim to serve. As such, this scoping review focuses specifically on ophthalmic image analysis AIaMDs that help inform clinical management and which have received regulatory approval in three jurisdictions with established regulatory pathways – Europe, Australia, and the United States of America (USA).

The study objectives were:

1. To identify ophthalmic image analysis AIaMDs with regulatory approval for clinical use in Europe, Australia, and the USA (covering all forms of market approval within that jurisdiction);

2. To describe the characteristics of these AIaMDs and the regulatory approvals granted to them;

3. To report and characterise the available evidence on model performance and clinical outcomes for these AIaMDs.

## Results

### Characteristics of eligible ophthalmic image analysis AIaMDs

Forty-four potentially eligible AIaMDs for ophthalmic image analysis were identified. Eight AIaMDs were excluded for the following reasons: they focused on image quality or denoising alone without impacting clinical care, were regulator-approved image management systems or platforms which may support AI models that are not themselves approved for commercial use (Supplementary Table 1), or were AI in a medical device (AIiMD) rather than AIaMDs. AIiMDs differ from AIaMDs in that they refer to AI as a component within a physical medical device, and may therefore include algorithms that primarily enhance physical hardware operations including image acquisition or device functionality, whereas AIaMDs represent AI software that functions as the medical device itself, which was central to the scoping review’s focus on algorithms for ophthalmic image analysis for clinical use.

In total, there were 36 eligible AIaMDs from 28 manufacturers. The 28 manufacturers were headquartered across a range of regions: Europe (11/28, 39%), Asia (6/28, 21%), the USA (6/28, 21%), Australasia (3/28, 11%), and the Middle East (2/28, 7%).

**Fig. 1:**
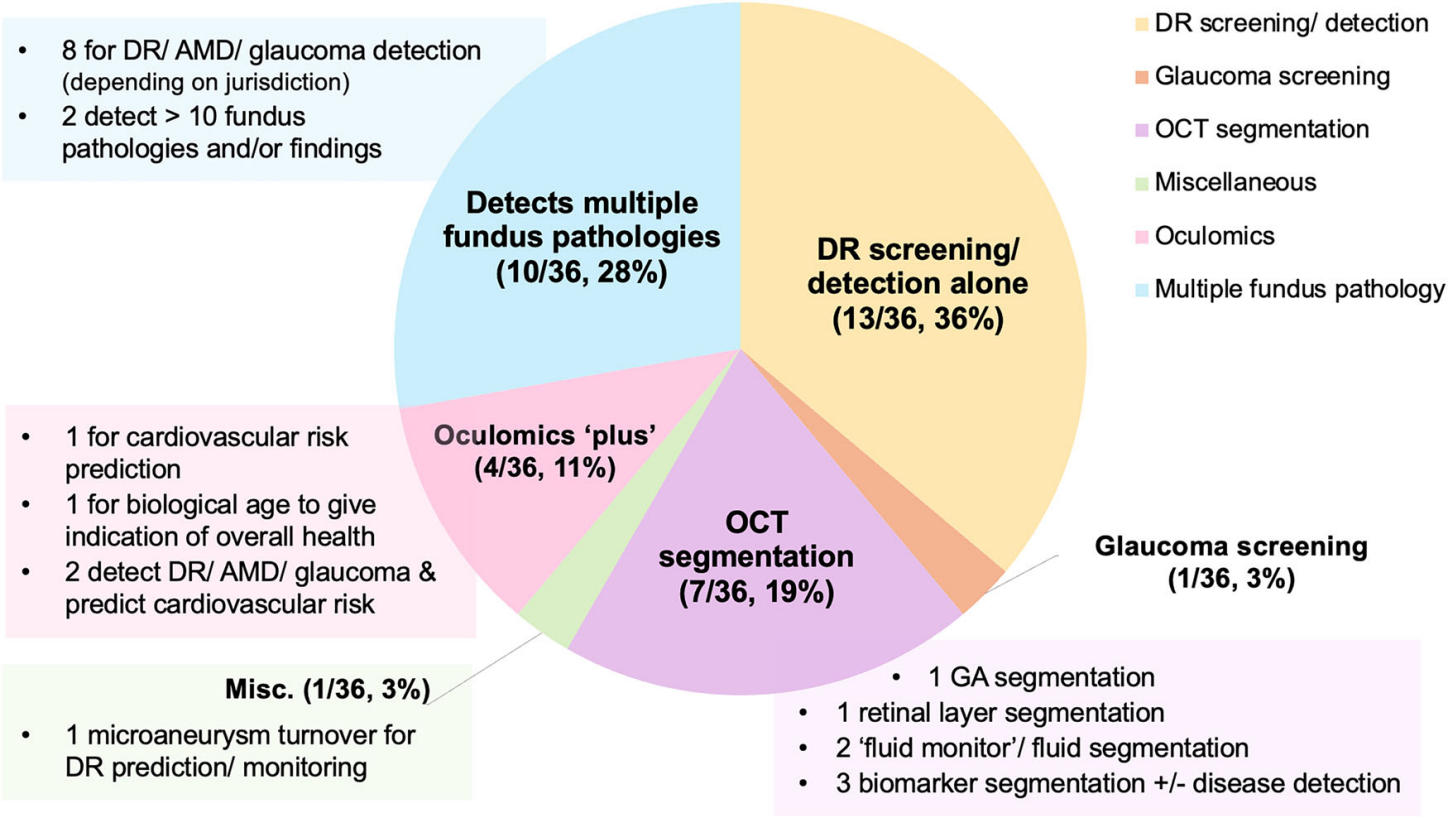
Task(s) performed by commercially available ophthalmic image analysis AIaMDs. This figure showcases the range of tasks performed by the 36 ophthalmic image analysis AIaMDs with regulatory approvals in the European Union, Australia, and the United States of America. These AIaMDs predominantly focus on the detection of posterior segment diseases, particularly DR. (AMD age-related macular degeneration, DR diabetic retinopathy, GA geographic atrophy, OCT optical coherence tomography).

In terms of task or intended purpose, 36% (13/36) were designed for diabetic retinopathy (DR) screening or detection alone. 28% (10/36) could detect multiple fundus pathologies—of these, eight focused on three common conditions (diabetic retinopathy (DR), age-related macular degeneration (AMD) and glaucoma, depending on the jurisdiction), one detected these conditions and nine other diseases, and one highlighted pathological findings and diseases on fundus images. One (3%) performed glaucoma screening alone. 19% (7/36) performed optical coherence tomography (OCT) segmentation for detecting or monitoring diseases and/or biomarkers. The remainder were designed for oculomics tasks (inferences about systemic health from via ophthalmic biomarkers, most commonly obtained through retinal imaging^13^) alone (2/36, 6%), oculomics tasks plus detection of DR, AMD, and glaucoma (2/36, 6%), or assessing microaneurysm turnover in DR to aid prediction and monitoring (1/36, 3%) (Fig. 1).

Input ophthalmic imaging modalities were either colour fundus photographs (CFP) (29/36, 81%) or retinal OCT scans (7/36, 19%). One AIaMD (ARDA, Verily Health) which was trained for DR screening using CFPs as inputs has also been used in ultrawidefield pseudocolour images as Optos AI (unable to confirm status of CE mark). For AIaMDs using CFPs, three were approved for clinical use or tested on images captured on handheld cameras - two on both tabletop and handheld devices (AEYE-DS, AEYE Health; SELENA + , EyRIS), and one on a handheld device only (Medios AI, Remidio). The remainder utilised a range of standard tabletop imaging devices. 58% (21/36) were paired with an image quality assessment system; the status of this was unclear in the remainder.

Deep learning models constituted the majority (29/36, 81%), of which 18 utilised convolutional neural networks and 11 did not specify the model architecture. Support vector machines represented a smaller proportion (2/36, 6%). For the remaining five AIaMDs, the model type could not be ascertained as this information was not provided by the manufacturer nor available publicly.

### Characteristics of regulatory approvals

**Fig. 2:**
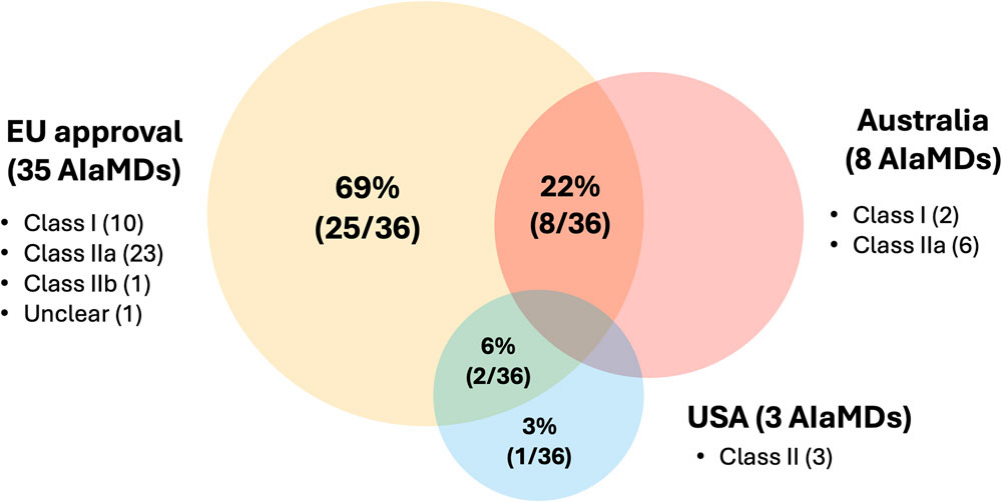
Regulatory approvals for ophthalmic image analysis AIaMDs across three jurisdictions. This figure shows the distribution of regulatory approvals for ophthalmic image analysis AIaMDs across one or more jurisdictions in the EU, Australia, and the USA, as well as the regulatory classes of AIaMDs in each jurisdiction. The majority of AIaMDs were approved for use in the EU, with a proportion of these also having been approved in a second jurisdiction. (EU European Union, USA United States of America, AIaMD Artificial intelligence as a medical device).

Almost all (35/36, 97%) were approved for use in the EU, and only 22% (8/36) and 8% (3/36) in Australia and the USA respectively (Fig. 2 and Table 1). 72% (26/36) were approved in a single jurisdiction—67% (25/36) in the EU alone and 3% (1/36) in the USA alone; the remainder were approved across two jurisdictions—the EU and USA (6%, 2/36), or the EU and Australia (22%, 8/36). None were approved across all three jurisdictions.

**Table 1:** Characteristics of commercially available ophthalmic image analysis AIaMDs and their regulatory approvals

AIaMD	Manufacturer	Manufacturer HQ	Imaging Modality	Task^a^	Regulatory Approvals (including year)^b^				Model Type	Image Quality
					Australia (TGA)	USA (FDA)	EU (EUDAMED)	UK (MHRA)^c^		
LumineticsCore (US) or IDx-DR (EU)	Digital Diagnostics Inc.	USA	Fundus photograph	Detects mtmDR (includes DMO)		Breakthrough device (2018) $\to$ Class II (2021, 2022)	CE Class IIa (2013)		Deep learning	Yes
Eyeart	Eyenuk, Inc	USA	Fundus photograph	Detects mtmDR and vision-threatening DR (including DMO)		510(k), Class II (2020, 2023)	CE Class IIb (2015)		Deep learning	Yes
Retmarker Screening or DAIRET (in Italy)	Retmarker (part of METEDA S.r.l.)	Portugal	Fundus photograph	Detects absence or presence of DR	Class IIa (2018)		CE Class IIa (2010)		Deep learning	Yes
RetmarkerDR Biomarker	Retmarker (part of METEDA S.r.l.)	Portugal	Fundus photograph	Tracks microaneurysm turnover to aid prediction of DR complications			CE Class IIa (NR)		NR	NR
SELENA+	eyRIS Pte. Ltd.	Singapore	Fundus photograph	Detects mtmDR (including DMO), referable/non-referable glaucoma suspect and referable/non-referable AMD	Class IIa (2023)		CE Class IIa (2020)		Deep learning	Yes
Automated Disease Assessment (ARDA)	Verily Life Sciences	USA	Fundus photograph	Detects DR and grades severity, detects DMO			CE Class IIa (2018)	Class IIa (2021)	Deep learning	Yes
Medios AI (or Medios DR)	Remidio Innovative Solutions Pvt. Ltd.	India	Fundus photograph	Detects referable DR including DMO			CE Class IIa (2023)	Class IIa (2021)	Deep learning	Yes
OphtAI	Evolucare/ ADCIS (partnership)	France	Fundus photograph	Detects DR (and grades severity), DMO, glaucoma, and AMD			CE Class IIa (2019)	Class IIa (2023)	Deep learning	Yes
RetCAD	Thirona Retina B.V.	Netherlands	Fundus photograph	Detects DR, AMD, and glaucoma, and grades severity of DR and AMD.	Class IIa (2020)		CE Class IIa (2022)	Class IIa (2022)	Deep learning	Yes
DeepDee	DeepDee	Netherlands	Fundus photograph	Detects DR, AMD, and glaucoma.			CE Class I (NR)		Deep learning	NR
MONA DR	MONA.health	Belgium	Fundus photograph	Detects referrable DR including DMO			CE Class I (NR)		Deep learning	Yes
MONA GLC	MONA.health	Belgium	Fundus photograph	Screens for glaucoma			CE Class I (2023)		Deep learning	Yes
Retinalyze	RetinaLyze System A/S (Ltd.)	Denmark	Fundus photograph	Detects DR, AMD, and glaucoma			CE Class I (2021)		Support vector machine	Yes
AEYE-DS	AEYE Health	Israel	Fundus photograph	Detects mtmDR (including DMO)		510(k), Class II (2022)			NR	NR
EyeCheckup	URAL TELEKOMÜNİKASYON SAN. TİC. A.Ş	Turkey	Fundus photograph	Detects mtmDR and vision-threatening DR (severe NPDR, PDR, DMO)			CE Class IIa (NR)		Deep learning	Yes
Reti-EyeReti-CVD (also known as DrNoon Fundus and DrNoon CVD)	Medi Whale Inc.	South Korea	Fundus photograph	*Reti-Eye:* Detects referrable retinal disease (DR, AMD, ERM etc.), glaucoma, and media opacities. *Reti-CVD:* Cardiovascular risk assessment	Class IIa (2021)		CE Class IIa (2021)	Class IIa (2022)	Deep learning	NR
ITOS Mass Screening	Voigtmann GmbH	Germany	Fundus photograph	DR screening: DR absent, suspicion of DR, DR present			CE Class IIa (2022)		NR	NR
EyeWisdom MCS/ Nexy AI	Visionary Intelligence Ltd.	China	Fundus photograph	Detects presence of 13 retinal diseases including: DR, wet and dry AMD, glaucoma			CE Class IIa (2024)		Deep learning	Yes
EyeWisdom DSS	Visionary Intelligence Ltd.	China	Fundus photograph	Detects presence of DR and grades severity			CE Class IIa (2022)	Class IIa (2021)	Deep learning	Yes
RetInSight Fluid Monitor	RetInSight GmbH $\to$ Topcon Healthcare Inc	USA	OCT macula	OCT segmentation and measurement of fluid-related biomarkers, to facilitate monitoring of nAMD			CE Class IIa (2022)	Class IIa (2022)	Deep learning	Yes
RetInSight GA Monitor	RetInSight GmbH $\to$ Topcon Healthcare Inc	USA	OCT macula	OCT segmentation and measurement of GA areas to facilitate visualisation and monitoring			CE Class IIa (2023)		Deep learning	NR
RetinAI Layer Segmentation	Ikerian (formerly RetinAI Medical AG)	Switzerland	OCT macula	OCT segmentation and measurement of retinal layers			CE Class IIa (2024)		Deep learning	NR
RetinAI Fluid Segmentation	Ikerian (formerly RetinAI Medical AG)	Switzerland	OCT macula	OCT segmentation and measurement of retinal fluid biomarkers			CE Class IIa (2024)		Deep learning	NR
RetinAI Macula Biomarkers	Ikerian (formerly RetinAI Medical AG)	Switzerland	OCT macula	OCT segmentation of macular biomarkers			CE Class IIa (2024)		Deep learning	NR
iPredict System	iHealthScreen Inc; Arif Systems	USA	Fundus photograph	*iPredict-DR*: Detects mtmDR or vision threatening DR *iPredict-AMD:* Detects referable AMD*iPredict-Glaucoma:* Detects glaucoma suspects	Class IIa (2022)		NR (2021)	Class IIa (2023)	Deep learning	Yes
TeleMedC DR grader	TeleMedC PTE LTD	Australia	Fundus photograph	Screens for DR, glaucoma, and AMD *indications vary according to jurisdictions	Class IIa (2021)		CE Class IIa (NR)		Deep learning	Yes
Eyetelligence system (Assure Plus)	Eyetelligence Pty Ltd; Optain	Australia	Fundus photograph	Screens for referable eye diseases including DR, glaucoma, and AMD. Additionally, screens for CVD risks	Class I (2021)		CE Class I (NR)		Deep learning	Yes
Eyetelligence system (Assure)	Eyetelligence Pty Ltd; Optain	Australia	Fundus photograph	Screens for referable eye diseases including DR, glaucoma, and AMD.	Class I (2019)		CE Class I (NR)		Deep learning	Yes
Diabetic Retinopathy Screening (DRISTi)	Artificial Learning Systems India Private Limited (Artelus)	India	Fundus photograph	Screens for the absence or presence of DR			CE Class I (NR)		Deep learning	NR
VUNO Med - Fundus AI	VUNO	South Korea	Fundus photograph	Identifies and locates the presence of 12 retinal abnormalities to support the diagnosis of retinal diseases			CE Class IIa (2020)	Class IIa (2023)	Deep learning	NR
BioAge	Toku Eyes	New Zealand	Fundus photograph	Determines biological age to give an indication of overall health			CE Class I (NR)	Class I (NR)	Deep learning	NR
CLAiR	Toku Eyes	New Zealand	Fundus photograph	Cardiovascular risk assessment		Breakthrough device designation	CE Class I (2024)	Class I (2024)	Deep learning	Yes
iGradingM or AutoGrader	Medalytix Group Ltd → National Services Scotland	UK	Fundus photograph	Detects absence or presence of DR (or whether image is ungradable)			CE Class I (2012)		Support vector machine	Yes
Altris AI	Altris	USA	OCT macula	OCT segmentation of retinal layers and biomarkers; detects retinal biomarkers and pathologies; measures segmentation volume and area; enables progression analysis			CE Class IIa (2019)		NR	NR
Ophthal	mr-doc	Italy	OCT macula	OCT segmentation of retinal layers and biomarkers to aid the monitoring of patients with DMO			CE Class IIa (2023)		Deep learning	NR
UMI DR	ULMA Medical Technologies	Spain	Fundus photograph	Detects absence or presence of DR			CE Class IIa (2023)		NR	NR

*AIaMD* artificial intelligence as a medical device, *AMD* age-related macular degeneration, *CVD* cardiovascular disease, *DMO* diabetic macular oedema, *DR* diabetic retinopathy, *GA* geographic atrophy, *mtmDR* more than mild diabetic retinopathy, *nAMD* neovascular AMD, *OCT* optical coherence tomography, *NR* not recorded.

^a^Based on regulatory documents where possible; otherwise peer-reviewed literature or manufacturer website.

^b^Incomplete availability of year of certification.

^c^Inferred from the MHRA’s regulatory database (PARD), which reports the manufacturer name but not that of the AIaMD.

While there is some variation in regulatory classification across the three jurisdictions, broadly speaking, the EU, ARTG (Australia), and FDA (USA) have three classes of medical devices, and the class assigned increases with the perceived risk level of the device. Class I AIaMDs pose the lowest risk to patient safety, and class III represents the highest risk. For AIaMDs approved for use in the EU, the majority were qualified as CE class IIa (23/35, 66%), followed by class I (10/35, 29%), and class IIb (1/35, 3%). For Australia, the AIaMDs were class IIa (6/8, 75%) or class I (2/8, 25%) only. All three regulatory approvals in the USA were class II.

The UK is a separate jurisdiction within Europe that accepts the CE mark. All 11 ophthalmic imaging AIaMDs registered on PARD (UK) were also approved for commercial use in the EU. These products had the same regulatory classes in both jurisdictions and have therefore not been considered separately.

Details on pivotal trials supporting regulatory approval were only available from summary documents on the FDA (USA) website; clinical evidence supporting regulatory approval was not available in the public domain for all other regulatory bodies.

### Study characteristics

The PubMed search identified 1164 studies, and manual searches (reference lists, correspondence with manufacturers, information on manufacturer websites) identified an additional 37 unique studies. Following de-duplication and abstract screening, 152 papers underwent full text review, resulting in 131 studies eligible for inclusion in the scoping review. The search strategy for each AIaMD is presented in Supplementary Table 1, and the PRISMA flow diagram for study selection in Supplementary Fig. 1.

Overall, the 36 AIaMDs were supported by 131 clinical evaluation studies (range 0-22, median 2, interquartile range (IQR) 1–6). Overall, 19% (7/36) of commercially available AIaMDs did not have published peer-reviewed evidence supporting their efficacy. 22% (8/36) of AIaMDs were supported by one validation study only.

In total, only 38% (50/131) of studies were conducted independently of the manufacturer. The remaining studies were directly funded by the manufacturer (14/131, 11%), were co-authored by researchers affiliated with the manufacturer (80/131, 61%), or both (81/131, 62%).

Model version was generally poorly reported across all studies (27%, 35/131). On a study-level, 22% (29/131) included comparisons of the AIaMD against human performance with no additional reference standard. Only 8% (10/131) of studies performed head-to-head comparisons of two or more AIaMDs. Sample size calculations were performed in 22% (29/131), of which five did not meet the required sample size.

Only 11 studies (8%) were interventional, meaning that the AIaMD impacted clinical care. Of these, three were post-deployment studies where data from routine clinical care was analysed retrospectively, and eight were experimental (seven non-randomised prospective studies, one RCT). These studies encompassed seven different AIaMDs with a DR screening use case; of these, two (iGradingM^14^ and Retmarker/ DAIRET^15^) have been deployed in the Scottish and Portuguese national DR screening services respectively for over a decade. The remaining studies were non-interventional, and were predominantly retrospective in nature (71/120, 59%). Distinguishing ‘silent’ trials (also known as translational trials) with certainty in this cohort was not always possible due to the ambiguous descriptions of study methodology in many cases.

These findings are summarised in Table 2.

**Table 2:** Clinical evidence for each AIaMD available in the peer-reviewed literature from our searches

AIaMD	Manufacturer	Highest Level of Evidence^a,b^	Head-to-head comparison (against other AIaMDs)	External validation	Post-market evidence
LumineticsCore (US) or IDx-DR (EU)	Digital Diagnostics Inc.	RCT, Prospective interventional	Yes	Multiple countries and settings	Yes
Eyeart	Eyenuk, Inc	Prospective interventional	Yes	Multiple countries and settings	No
Retmarker Screening or DAIRET (in Italy)	Retmarker (part of METEDA S.r.l.)	Post-deployment	Yes	Three countries	Yes
RetmarkerDR Biomarker	Retmarker (part of METEDA S.r.l.)	Retrospective	No	Single country	No
SELENA+	eyRIS Pte. Ltd.	Prospective silent	No	Four countries	No
Automated Disease Assessment (ARDA)	Verily Life Sciences	Prospective interventional	No	Four countries	No
Medios AI (or Medios DR)	Remidio Innovative Solutions Pvt. Ltd.	Prospective silent	Yes	Two countries	No
OphtAI	Evolucare/ ADCIS (partnership)	Retrospective	No	No	No
RetCAD	Thirona Retina B.V.	Prospective observational	No	Four countries	No
DeepDee	DeepDee	Not available	N/A	Not available	No
MONA DR	MONA.health	Retrospective	No	Single country	No
MONA GLC	MONA.health	Retrospective	No	Multiple countries	No
Retinalyze	RetinaLyze System A/S (Ltd.)	Prospective observational	Yes	Two countries	No
AEYE-DS	AEYE Health	Prospective observational	Yes	Single country	No
EyeCheckup	TELEKOMÜNİKASYON SAN. TİC. A.Ş	Prospective observational	No	Single country	No
Reti-EyeReti-CVD	Medi Whale Inc.	Prospective observational (Reti-Eye); Retrospective (Reti-CVD)	No	Four countries	No
ITOS Mass Screening	Voigtmann GmbH	Not available	N/A	Not available	No
EyeWisdom MCS/ Nexy AI	Visionary Intelligence Ltd.	Prospective observational	Yes	Single country	No
EyeWisdom DSS	Visionary Intelligence Ltd.	Prospective observational	Yes	Single country	No
RetInSight Fluid Monitor	Topcon Healthcare Inc.	Retrospective	No	Multiple countries	No
RetInSight GA Monitor	Topcon Healthcare Inc.	Retrospective	No	No	No
RetinAI Layer Segmentation	Ikerian (formerly RetinAI Medical AG)	Retrospective	No	Single country	No
RetinAI Fluid Segmentation	Ikerian (formerly RetinAI Medical AG)	Retrospective	No	Single country	No
RetinAI Macula Biomarkers	Ikerian (formerly RetinAI Medical AG)	Retrospective	No	Single country	No
iPredict System	iHealthScreen Inc; Arif Systems	Prospective observational	Yes	Three countries	No
TeleMedC DR grader	TeleMedC PTE LTD	Prospective observational	No	Three countries	No
Eyetelligence system (Assure Plus)	Eyetelligence Pty Ltd; Optain	Prospective observational	No	Three countries	No
Eyetelligence system (Assure)	Eyetelligence Pty Ltd; Optain	Prospective observational	No		No
Diabetic Retinopathy Screening (DRISTi)	Artificial Learning Systems India Private Limited (Artelus)	Not available	N/A	Not available	No
VUNO Med - Fundus AI	VUNO	Retrospective	No	Four countries	No
BioAge	Toku Eyes	Retrospective	No	No	No
CLAiR	Toku Eyes	Retrospective	No	Single country	No
iGradingM or AutoGrader	Medalytix Group Ltd → National Services Scotland	Post-deployment	No	Two countries	Yes
Altris AI	Altris	Not available	N/A	Not available	No
Ophthal	mr-doc	Not available	N/A	Not available	No
UMI DR	ULMA Medical Technologies	Not available	N/A	Not available	No

This table presents the best available evidence identified from our search of the peer-reviewed literature in July 2024. The availability and level of evidence are presented, but the quality and methodological rigour of this evidence is not assessed (out of scope).

^a^‘We have used the following definitions for study types:

Prospective observational study: Clinical data are collected prospectively, which allows for subsequent retrospective evaluation of AIaMD performance on prospectively gathered data.

Prospective silent trial (also known as shadow, translational trial): The AIaMD is run in real time on live data, but its predictions are not visible to clinicians and do not affect patient care. The goal is to assess how the model performs in the target clinical environment, simulating deployment without clinical impact.

Prospective interventional study: The AIaMD is prospectively deployed with outputs shown to clinicians, who may incorporate them into care decisions. This design evaluates how the AI affects clinical workflows, behaviour, and potentially patient outcomes.

Randomised controlled trial (RCT): a type of prospective interventional study wherein patients, clinicians, or clinical episodes are randomised to either an AI-assisted arm (where AI output informs care) or a control arm (usual care) to evaluate causal impact.

Post-deployment monitoring: Ongoing surveillance after regulatory approval and integration of an AIaMD into routine clinical practice.

^b^Distinguishing ‘silent’ trials with certainty in this cohort was not always possible due to the ambiguous descriptions of study methodology in several instances. In such cases, we have inferred the methodology from the available evidence provided, and have adopted a conservative approach in doing so.

*AIaMD* artificial intelligence as a medical device.

### Dataset characteristics

**Fig. 3:**
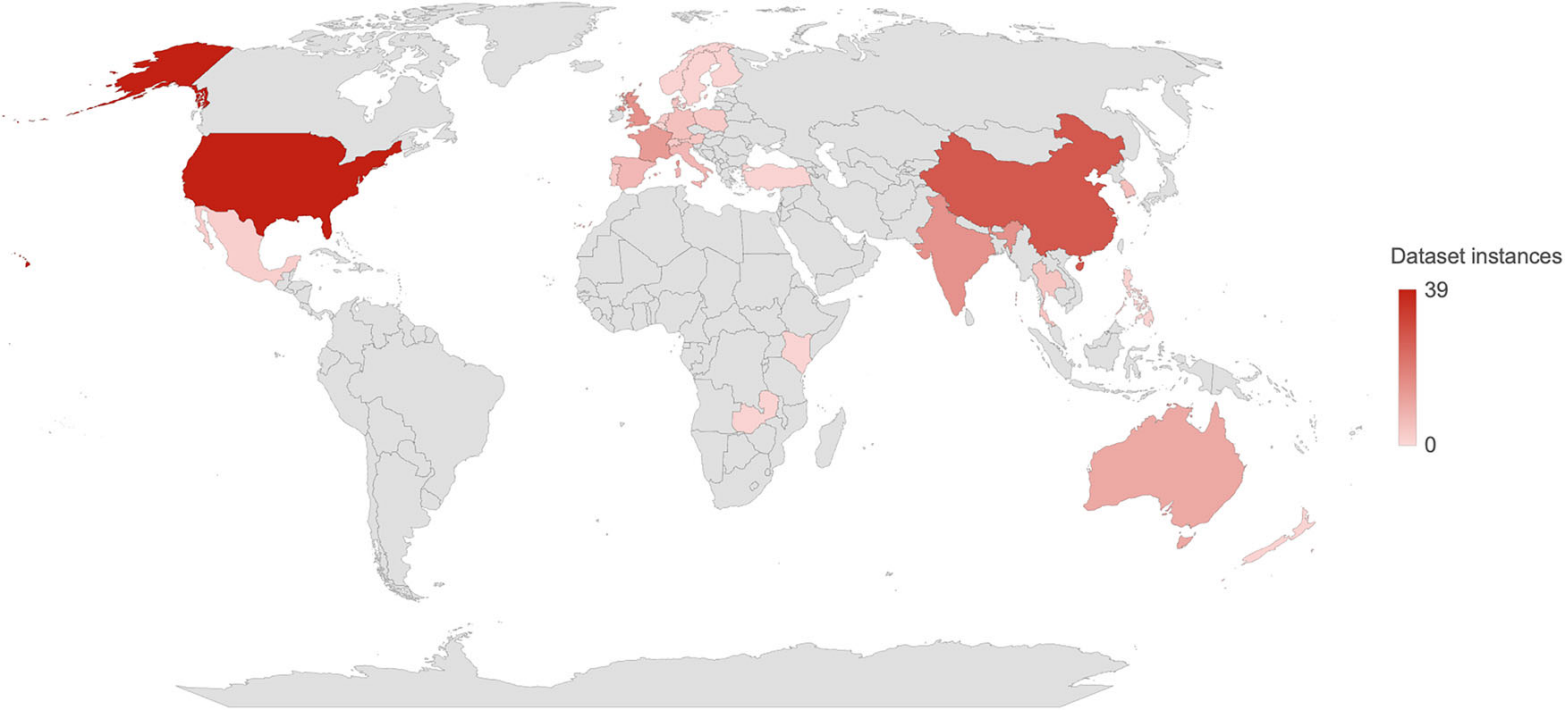
Geographical distributions of datasets. This figure shows the geographical distributions of dataset instances used in validation studies of ophthalmic image analysis artificial intelligence as a medical device commercially available in Europe, Australia, and the United States of America (USA). The USA and China were the most common sources.

The 131 studies described 192 datasets or patient cohorts across 31 countries, most commonly the USA (39), China (27), the UK (15), India (15), France (13), and Singapore (13) (Fig. 3). 25% (48/192) of the datasets were from low- and middle-income countries (LMICs) (based on the World Bank’s Classification)^16^. The datasets were mostly from multiple sites (107/192, 56%).

Dataset size ranged from 19 to 30,000 patients for datasets where the numbers of patients were reported; this could not be summarised due to the heterogeneity of the unit of reporting (patient, visit, eye, or image). Demographic subgroups were poorly reported across the 192 datasets—age was reported in 52% (101/192), sex in 51% (97/192), and ethnicity in 21% (40/192). Study duration (or duration of data collection) was reported in 54% (103/192) only.

45% (87/192) of the datasets used for validation were from a range of publicly available datasets with different levels of data accessibility^17^, such as Messidor/ Messidor-2 (8 instances); datasets from pre-existing epidemiological studies such as the Singapore Epidemiology of Eye Diseases study (8 instances), AREDS study (3 instances), or the UK Biobank (3 instances); or landmark RCTs such as the HARBOR trial (2 instances), or the HAWK, HARRIER, and FILLY trials (1 instance of each).

### Reference standard setting

Reference standard setting was variable. For the 167 datasets used to evaluate AIaMD diagnostic accuracy, reference standards were typically determined by experienced human graders grading the same image used as inputs for the AIaMD, although a small subset used the findings from routine clinical care (e.g. dilated fundus examination), or different imaging protocols (e.g. 7-field ETDRS or 4-wide field photography protocol for DR screening), or both, as the reference standard. Datasets were labelled by one grader (29/167, 17%), two graders (43/167, 26%), three or more graders (41/167, 25%), or not specified in the remainder.

The approach to adjudication varied considerably as well. Single grader studies did not require adjudication, although some elected to adjudicate those cases where the AIaMD and the human grader disagreed. For disagreements between two or more graders, many did not require additional adjudicators, instead opting for consensus discussion, a majority voting rule, or re-review in a round robin fashion until consensus was achieved. Others sought the input of an additional senior clinician to arbitrate.

The majority (84%, 141/167) provided some description of the graders involved in setting the reference standard, predominantly by stating the profession (e.g. ophthalmologist, retinal specialist, non-ophthalmologist grader). The graders’ level of experience was not well characterised overall, with many citing “trained graders”, “experienced graders”, or “experts”, without elucidating the number of years of experience or familiarity with the specific task.

## Discussion

This scoping review has identified and described the characteristics of ophthalmic image analysis AIaMDs with regulatory approval for clinical use. The available evidence for the effectiveness of these AIaMDs has also been curated and characterised.

Thirty-six ophthalmic image analysis AIaMDs with regulatory approvals in Europe, Australia, and the USA were identified. They serve four main intended uses: detection or screening of 1) DR or 2) DR and other fundus pathologies, 3) OCT segmentation for biomarker and/or disease detection or progression, and 4) oculomics tasks.

The heavy emphasis on DR aligns with a significant public health need, given that DR is a leading cause of preventable blindness in the working age population, and early detection and intervention can reduce the risk of vision loss^18^. As diabetes becomes more common globally, there is an opportunity for AIaMDs to help improve the scalability and efficiency of screening processes, alleviate some of the burden on healthcare systems, and improve access to care. Existing national or regional DR screening programmes lend themselves well to AI integration due to their standardised nature and pre-existing quality assurance frameworks, particularly as many mandate double-reader screening for a subset of cases^19^.

However, there is significant scope for expanding AIaMD applications to other imaging modalities, ocular conditions, and use cases as well. In particular, there is rising interest in further oculomics applications to detect or predict the risk of chronic systemic diseases with a significant morbidity and mortality burden, including neurodegenerative diseases such as Alzheimer’s dementia or Parkinson’s disease^20^. Tools such as target product profiles, which are well-established in other fields and are in development for AIaMDs^21^, can guide product development and evaluation by laying out the requirements necessary for successful implementation. This may help accelerate the development of AIaMDs that align with stakeholders’ needs.

It is also important to note how the interplay between regulatory approval, development costs, and reimbursement structures may shape the commercialisation strategies for AIaMDs, affecting both their availability and the scope of applications pursued by manufacturers^22^. Notably, nearly all 36 AIaMDs were commercially available in the EU, but only three were approved for use in the USA. This discrepancy is likely to be multifactorial. We speculate that key contributors may include the varied value propositions and reimbursement structures for tools across different healthcare systems, as well as differing regulatory frameworks across jurisdictions^23,24^.

For example, the clinical evidence requirements appear to differ substantially - several EU MDR approvals have been based on retrospective observational data, whereas all three FDA-authorised ophthalmic imaging AIaMDs to date have been supported by prospective multicentre pivotal trials, which has obvious time and financial implications. This contrast may stem from the FDA’s stricter ‘De Novo pathway’ for “first-in-class” devices. Unlike their standard 510(k) pathway, this stipulates more rigorous clinical evaluation to demonstrate safety and effectiveness for novel technologies. IDx-DR/LumineticsCore (Digital Diagnostics) was cleared via this pathway^25^. EyeArt (EyeNuk) designed an equivalent pivotal trial which proceeded before the IDx-DR predicate was approved, followed by AEYE-DS (AEYE Health), both of which secured 510(k) clearance based on substantial equivalence to the IDx-DR predicate. Notably, the FDA has designed a “Breakthrough Device Designation” pathway to expedite regulatory review and facilitate increased regulator interaction and support with commercialisation for eligible devices, potentially leading to faster market access and hence patient benefit, over conventional pathways^26^. IDx-DR/LumineticsCore (Digital Diagnostics) has previously benefited from this, and another (CLaiR, Toku Eyes) has latterly received this designation. This pathway was established in 2015 but does not appear to have contributed significantly to addressing the discrepancy, suggesting that market factors may play a more significant role. For example, the EU market comprises multiple different healthcare systems with diverse reimbursement models, whereas AIaMDs in the USA must secure reimbursement through Medicare, a process that can be particularly challenging in a fee-for-service paradigm. Future work should consider qualitative research to elucidate the true underlying reasons for these differences, and to consider how the regulation of AI health technologies can balance safety and maximising patient benefit.

This study found that many clinical validation studies were predominantly or solely conducted on existing datasets. These included retrospective open access datasets, epidemiological studies, and data repurposed from previous RCTs, which tend to have strict eligibility criteria and may not reflect real-world practice settings. It has previously been reported that publicly available ophthalmic imaging datasets tend towards inadequate reporting of basic demographic characteristics (age, sex, ethnicity), disparities in representation of different population and disease groups, and uneven geographical distributions, highlighting issues of health data poverty that may encode biases into AI models^17,27^. In addition, their differing disease prevalence and relatively high image quality may not reflect real world clinical practice, potentially affecting their suitability for robust clinical evaluation of AIaMDs. To mitigate this, future validation studies should consider the STANDING Together (STANdards for data Diversity, INclusivity, and Generalisability) recommendations for documenting and using health datasets in developing and testing AI health technologies^28^, as well as model cards or similar initiatives that encourage transparency of model reporting, including details on training datasets where feasible, to enhance accountability and mitigate biases while respecting proprietary constraints^29^.

In addition, we demonstrate that the evidence base for ophthalmic image analysis AIaMDs with regulatory approvals remains heavily weighted towards retrospective and observational studies. This mirrors findings from a 2021 review of FDA-authorised AIaMDs, which found that few regulatory submissions reported prospective data^30^. While leveraging large retrospective datasets is resource- and cost-effective, it has become increasingly recognised that this is merely an initial step, and that AI deployment requires a sociotechnical approach to inform safe integration into current clinical workflows^31^. Testing the fragility of AIaMD performance in prospective implementation-focused trials (either silent or interventional) is essential to identify challenges that may not be apparent in silico, and may help drive improvements in model design, training, and deployment strategies^32,33^. This is particularly important for AIaMDs that are intended for use as clinical decision support tools, in which incorporation and evaluation of human-computer interaction is essential.

Our review found that few studies of commercially available AIaMDs examined their performance in a real-world clinical workflow. There was significant variation in the number and depth of validation studies across the AIaMDs under study. IDx-DR/LumineticsCore (Digital Diagnostics Inc.) exemplifies high quality evidence, with external validation across a wide range of countries, population groups, and study types demonstrating real-world clinical effectiveness. Beyond diagnostic performance, this AIaMD has been tested in a RCT demonstrating improved adherence to follow-up compared to traditional referral routes^34^. Post-deployment studies have also demonstrated the utility of AI-driven point-of-care screening in improving patient access to DR screening and closing the health equity gap^35^, while also improving ophthalmology follow-up rates for patients with referrable DR, potentially by reducing the time taken to receive their screening results^36^. While RCTs are the gold standard for generating evidence in many fields of medicine, whether they are necessarily the best method of validating AIaMDs’ safety and effectiveness remains to be determined, given that the problems AIaMDs address often lack a reference standard, and human-computer interactions and explainability issues may limit replicability and reproducibility. At the very minimum, moving beyond diagnostic accuracy metrics to real-world evidence will be instrumental in making the case for real-world deployment and integration into the clinical workflow. Future studies should consider patient-centred outcomes (e.g. adherence to follow-up, patient satisfaction, equity of access, health-related quality of life measures), workflow-related metrics (e.g. time to diagnosis, referral appropriateness, clinician workload), health system and economic outcomes (e.g. cost-effectiveness, impact on service delivery), and implementation outcomes (e.g. user experience, ease of integration into existing infrastructure, training needs). This will be critical to evaluate the real-world effectiveness, safety, and the feasibility of deploying AIaMDs at scale. These outcome domains should be tailored based on the specific AIaMD’s intended use case, stakeholder needs, and healthcare setting.

This is important because while regulatory clearance is an essential first step to ensure that AIaMDs function safely, it does not necessarily guarantee consistent real-world performance across diverse clinical settings. Published guidelines for AIaMD implementation processes consistently highlight initial pre-implementation local evaluations and post-deployment monitoring as best practices^37,38^. Methods to deliver these local forms of evidence vary, but they all aim to help adopters to address the limited generalisability of AIaMD evidence between settings and over time. Examples include recurring local validation to monitor performance within their specific patient population, clinical workflow, and technical infrastructure over time^39^, or performing medical algorithmic audits to systematically evaluate and pre-empt AI errors and patient harms^40^.

One-fifth of ophthalmic image analysis AIaMDs did not have publicly available peer-reviewed evidence supporting their effectiveness. This does not necessarily equate to an absence of evidence, as some manufacturers choose not to publish results of studies submitted to regulatory bodies or conferences. However, this raises important questions about the incentives for manufacturers to invest in, conduct, or publish rigorous studies on their AIaMDs beyond regulatory requirements, particularly given the significant financial, logistical and time costs^41^, especially for small and medium-sized enterprises with limited resources. Without strong incentives—whether regulatory, financial, or reputational—manufacturers may not necessarily prioritise evidence generation for real-world deployment. This pushes the due diligence on to cross-functional AI adopter teams, which may have varying levels of resources and different processes for obtaining and critically appraising this evidence.

Alternatively, evidence of AIaMD performance can be generated independently of the manufacturer, either by facilitating participation in research led by academic institutions or conducting post-deployment studies. This can be helpful in providing objective evidence of performance, but was only the case for one-third of studies identified. For example, in three researcher-led head-to-head comparison studies of multiple AIaMDs, several manufacturers either did not respond to enquiries or ultimately withdrew from participation, citing commercial or unspecified reasons^42–44^.

Facilitating greater transparency from manufacturers is a key step in building trust in AIaMDs among stakeholder groups. Possible strategies could include regulatory mandates for public disclosure of clinical evidence from development through to post-market surveillance (particularly for more mature AIaMDs)^45^, supported by additional funding, which could help align AI development and deployment with the ethical imperatives of safety, inclusivity, and equity. Conducting this scoping review has surfaced several challenges in navigating regulatory databases due to limited access and/or search functionality, data fragmentation, and a dearth of useful information. This presents a real challenge to healthcare provider organisations considering AI implementation, who are unlikely to have the resources or expertise to identify all regulated AIaMDs that may meet their needs. To mitigate this, establishing public-facing databases could facilitate stakeholder access to information about available products, their performance, and safety risks^46^. This approach has been led by the field of radiology, with examples such as the Health AI Register listing regulator-approved AI products^47^, or the Royal College of Radiologists’ AI registry featuring AIaMDs being deployed or tested in the UK^48^. Other groups have developed an open-access database summarising information about FDA-approved AIaMDs^49^. National or international registries, for example through a federated registration approach^50^, could also help standardise the reporting and evaluation of AIaMDs, and ensure that information is accessible, consistent, and reliable to inform successful implementation.

This study identified significant variability in reference standard setting, in terms of the number and experience level of graders as well as the arbitration process. Image-based reference standards are subjective by nature, and interpretation may sometimes differ even between experts, potentially leading to inconsistencies in the labelling and ground-truthing process^51,52^. Any variation in the reference standards against which AIaMDs are evaluated can influence performance metrics and affect the perceived effectiveness of these models^53^. To address this, researchers should consider increasing the number and experience of graders required, and ensure an unbiased arbitration process, all while carefully balancing the trade-off between the quality of labelling and the resources required^54^. In any case, transparency in this process is a valuable safety mechanism, but information on reference standard setting was not always clearly documented in the studies identified.

Another key consideration is whether and how AIaMD performance may be influenced by the imaging device used. Differences in hardware may produce variations in image resolution, size, field of view, and quality. Several AIaMDs identified in our searches have reported differences in model performance across some types of cameras used to capture colour fundus images^55,56^, which was not necessarily the case across all AIaMDs^57^. For other modalities such as OCT scans, re-training AI models may be necessary to optimise performance in devices from other manufacturers^52^. This may of course vary depending on the diversity of training data for each model. Nevertheless, ensuring that AIaMDs are robust across imaging devices from different manufacturers would benefit from extensive testing with diverse datasets. In addition, re-validation (with or without re-training) is essential to optimise AIaMD performance in new devices, and aligns with regulatory requirements, such as the FDA’s mandate to validate and re-certify each new device to ensure full regulatory compliance. However, this does not appear to be a mandatory requirement for the EU and Australia. Notably, the intended use statements for FDA-approved AIaMDs such as IDx-DR/LumineticsCore, EyeArt, and AEYE-DS specify the imaging device(s) with which they are allowed to be used. This was not the case for the EU and Australia. The imaging device used was not always well-documented in the validation studies we identified as well.

Oculomics is an emerging field, as evidenced by the four AIaMDs with regulatory approvals that we have identified. However, performing clinical validation for such AIaMDs may pose unique challenges. These models differ from standard diagnostic AI models in several key aspects, such as the need to handle more diverse and complex data types, including multimodal data combining ophthalmic imaging, systemic information or imaging, and/or genomic data. In addition, demonstrating the ability to predict a range of systemic conditions that may not have well-defined clinical endpoints (e.g. the presence or absence of a specific disease) renders establishing a ground truth more difficult. Additionally, they require integration with diverse clinical workflows in other fields beyond ophthalmology. The potential for these models to reveal previously unknown associations between ocular and systemic health raises questions about clinical interpretability, generalisability, and ethical considerations as well.

Several challenges were encountered in the conduct of this scoping review, which highlight broader issues in the landscape of AIaMD evaluation.

A substantial proportion of manufacturers (18/28, 64%) did not respond to requests for further information or clarification on their AIaMD(s). To mitigate this, the missing data was supplemented with publicly accessible sources wherever possible, and multiple methods of corroboration were employed, including conducting searches of manufacturers’ websites, evaluating peer-reviewed publications, and internet search engines. It is important to highlight that only the FDA has made a summary of regulatory documents publicly available for each AIaMD—this was not the case for the other regulatory agencies. The findings presented in this review are therefore contingent upon the quality and availability of data from these pragmatic methods, and reflect the most accurate information obtainable under these constraints. This is also likely to be the same evidence that decision-makers are presented with to decide on procurement.

The search functionality of the databases was not well suited to identifying software medical devices with and without AI components, particularly class 1 devices, for which registration on EUDAMED is not currently mandated. The scope of this review also excluded AIiMDs (as opposed to AIaMDs) as there were no means to construct a search strategy with meaningful sensitivity for such approvals in current regulatory databases. As such, the two hardware/software ‘system’ products with AI components for ophthalmic image analysis, SCANLY home monitoring (Notal Vision Inc.) and EyeLib (MIKAJAKI SA), which were identified through separate searches, were therefore not included.

Several studies did not explicitly name the AIaMD they were evaluating. This omission made determining the relevance of a given paper challenging on occasion, and a pragmatic approach in assessing eligibility was therefore taken. Some AIaMDs also undergo name changes across versions, or are marketed under different names in various jurisdictions. For example, the AIaMD originally known as the Iowa Detection Program was rebranded commercially as IDx-DR and subsequently LumineticsCore (depending on the jurisdiction). As these devices transition from academic to commercial products, clear documentation of naming as well as versioning would facilitate future research such as comparative studies and systematic reviews. Tracking the specific version of the AIaMD used in each study is also essential for assessing performance, particularly when updates or retraining could significantly impact clinical outcomes. Unfortunately, this information was frequently poorly recorded in the studies reviewed. This is a requirement of the CONSORT-AI extension^58^ reporting guideline for RCTs involving AI models, and should be considered for other types of validation to improve transparency and replicability.

The scoping review had an Anglocentric focus by design, and included only AIaMDs with regulatory approvals across three jurisdictions: Australia, Europe, and the USA. This was a pragmatic choice given that these jurisdictions possess centralised regulatory databases that facilitated our search process (albeit with certain limitations in their search functionalities and level of transparency), are members of the International Medical Device Regulators Forum, and have a well-established history of authorising AIaMDs for their markets. Exploring regulatory approvals in other regions such as Asia, South America, or the Middle East would offer valuable insights, especially considering the rapid advancements in AI health technologies there. Notably, major players such as China’s National Medical Products Administration (NMPA) have introduced guidelines that classify and designate AIaMDs according to risk, while setting forth standards for data security, validation and streamlined regulatory submissions^59^. Similarly, India’s Central Drugs Standard Control Organisation (CDSCO) is working to formulate its regulatory stance on AI-driven tools. Future research could aim to explore regulatory approvals in these major markets via alternative data sources or collaborating with local experts to systematically map AIaMD development, so as to illuminate disparities in regulatory infrastructure, identify best practices, and guide more harmonised oversight of AIaMDs.

Finally, this study focused on peer-reviewed publications identified through PubMed searches only, omitting evidence that exists only in preprints or conference abstracts. This was a pragmatic decision aimed at ensuring the reliability and scientific rigour of the included studies. In addition, some manufacturers may opt to submit evidence directly to regulatory bodies without pursuing publication in peer-reviewed journals, which would lead to underrepresentation in the academic literature, which is an inherent limitation of the current regulatory process.

In summary, a growing number of ophthalmic image analysis AIaMDs have passed regulatory approval for clinical use globally, though availability varies substantially between jurisdictions and identifying them can be challenging. These AIaMDs predominantly focus on the detection of posterior segment diseases from CFPs, particularly DR. There is scope for expanding AIaMD applications to other imaging modalities, ocular conditions, and use cases. Greater emphasis should be placed on accurate and transparent reporting of datasets to highlight risks of varied subgroup performance; this is critical to ensuring equitable performance as some populations may be underrepresented in the training data. The evidence available to evaluate the effectiveness of individual AIaMDs is extremely variable, with a focus on retrospective diagnostic accuracy study designs, but limited data on outcomes related to real-world implementation. A requirement for more high-quality prospective implementation studies may help promote transparency and confidence in performance for end-users. Finally, regulatory frameworks for AIaMDs may benefit from a more standardised approach to evidence reporting. This could provide clarity for manufacturers as they plan their clinical evaluation strategies, and provide potential adopters with more of the information they need to make responsible choices about AI innovation.

## Methods

In line with the primary objectives of this study, a scoping review was selected in preference to a systematic review. This was because our purpose in conducting this review was to identify relevant AIaMDs for ophthalmic image analysis and map the available evidence for effectiveness to identify research gaps, instead of providing an unbiased and precise effect estimate^60^.

### Protocol and registration

The review adheres to the PRISMA-ScR (Preferred Reporting Items for Systematic Reviews and Meta-Analyses extension for Scoping Reviews)^61^ framework where applicable. The protocol was registered at https://osf.io/cmkyv and published prior to full execution^62^. The methodology is summarised below.

### Eligibility

The review focused on AIaMDs using ophthalmic image analysis to help inform clinical management, which have regulatory approvals in the USA, Australia, and Europe. No restrictions were placed on the type of imaging modality or the intended use. AIaMDs were defined as having a partial or fully data-led mechanism, rather than an exclusively rule-based mechanism^63^.

With regards to the evidence underpinning each AIaMD, only primary research evaluating performance in human participants was included. Eligible study types included randomised controlled trials (RCT), non-randomised interventional studies, ‘silent’ trials, or retrospective observational studies. Systematic reviews and meta-analyses, case series, case reports, commentaries, and expert opinions were not eligible. No date or language restrictions were applied to the electronic search. Only peer-reviewed publications were considered. Preprints and conference abstracts were ineligible.

### Search strategy and sources of information

To identify potentially eligible AIaMDs, the following regulatory databases were searched: the Food and Drug Administration (FDA, USA) database, the Australian Register of Therapeutic Goods (ARTG, Australia), the Public Access Registration Database (PARD, United Kingdom), and the European Database on Medical Devices (EUDAMED, European Union (EU)). A tailored search strategy was designed to circumvent the challenges inherent in navigating existing regulatory databases (such as limited search functionality, transparency, and lack of AI-specific global medical device nomenclature limiting identification); this involved an exhaustive review of the product class codes and predicate devices (if applicable) with which each known eligible device was associated. The search commenced with a list of 15 AIaMD for ophthalmic imaging. This represented the sum of the authors’ awareness of regulated products at the start of the search process and a pragmatic search of relevant academic literature^64^. This strategy was adopted due to limitations in the search functionality of these regulatory databases. No AI tools were used in the search process.

Following confirmation of AIaMD eligibility, PubMed was systematically searched up to 24 July 2024 for publications relevant to each AIaMD and its manufacturer by combining both search terms with an “OR” Boolean operator. Where appropriate, these searches were limited to relevant ophthalmology-specific studies using relevant key terms, for example “retin*” for AIaMDs relating to diabetic retinopathy. The search terms and number of hits are presented in the supplementary materials (Supplementary Table 2). Manual searches of reference lists and manufacturers’ websites were also conducted to identify additional peer-reviewed publications.

The manufacturers of all eligible AIaMDs were contacted with a standardised email template (Supplementary Table 3) to provide clarification, corroboration, and/or additional peer-reviewed publications not identified in earlier searches. Three attempts were made to contact each manufacturer. This additional step was undertaken to help ensure that the data captured were as comprehensive as possible. Preliminary scoping searches had highlighted some areas of ambiguity, including instances where studies did not specify the name of the AIaMD or manufacturer, or cases where devices underwent a name change from one version to the next. Information on all eligible AIaMDs was also collated from relevant publications identified from the above searches, and supplemented using an internet search engine (Google Search, Google).

### AIaMD and study selection

Two authors (AK/AYO, HDJH) searched the regulatory databases independently and screened all identified AIaMDs for eligibility. Any disagreements were discussed, and if consensus could not be reached, these were resolved with recourse to another author (ED) for arbitration. In instances where an AIaMD’s eligibility or its regulatory approval status could not be determined with publicly available evidence, the manufacturers were contacted to seek clarification (AYO). If no response was forthcoming, the ambiguity about the AIaMD’s eligibility and the rationale for including or excluding were duly recorded.

Each title and abstract from the PubMed search were screened independently by two review authors (AYO and PT/MS) to determine eligibility. Full-text articles were reviewed according to the eligibility criteria set out in the protocol. At each stage, results were compared and consensus reached, with arbitration by a third reviewer (HDJH) as required.

### Data extraction

Data extraction was undertaken by AYO (and verified by PT/ MS) in two phases, using standardised data extraction forms designed and piloted for the purposes of this review.

Phase 1: The following outcomes were extracted for each eligible AIaMD:

- Jurisdiction in which regulatory approval was given.
- Class assigned under FDA, TGA, UK MDR (Medical Devices Regulations 2002) and/or EU MDR (Regulation (EU) 2017/745) or MDD 93/42/EEC.
- Intended use statement (IUS) (or manufacturer’s description of purpose when IUS was not available.
- Ophthalmic imaging modality.
- AI model type and architecture.

Phase 2: The following outcomes were extracted for each eligible study:

- Study information: title, author name, publication status, funding source, conflicts of interest, author affiliations with manufacturers.
- Study methodology and outcomes: study duration, study design, internal/external validation, reference standards, comparison between AIaMD and humans, AIaMD version etc.
- Data set or cohort details: source of dataset, size of dataset or number of participants, setting, number of countries, number of centres, and participant demographics (age, gender, ethnicity).

### Data synthesis

The data for each AIaMD were synthesised to give an overview of its characteristics and that of its regulatory approval(s) through narrative and tabular approaches. Study- and cohort-level data were similarly synthesised and presented using descriptive statistics to outline the characteristics of the included studies.

### Differences from the protocol

Two changes were made to the published protocol^62^. Firstly, although a quality assessment of eligible studies was initially planned, it was later determined that this did not align with the stated purpose of the scoping review, which sought to map the evidence for commercially available ophthalmic image analysis AIaMDs. Secondly, extracting data on model performance was not carried out for similar reasons; the heterogeneity of AI models (even within the same use case), study types, study settings, populations, and technical factors (such as camera types) limited the feasibility and value of meta-analysis, even at the level of individual AIaMDs.

## Supplementary information

Checklist

Supplementary Materials

## Data Availability

No datasets were generated or analysed during the current study.

## Abbreviations

AIaMD: Artificial intelligence as a medical device
AIiMD: AI in a medical device
AMD: Age-related macular degeneration
ARTG: Australian Register of Therapeutic Goods
DR: Diabetic retinopathy
EU: European Union
EUDAMED: European Database on Medical Devices
FDA: Food and Drug Administration
OCT: Optical coherence tomography
PARD: Public Access Registration Database
RCT: Randomised controlled trial

## Code availability

Not applicable as no code was generated.
