# Supplementary material for: A scoping review of artificial intelligence as a medical device for ophthalmic image analysis in Europe, Australia and America
Source: NPJ Digit Med. 2025 May 29;8:323. doi: 10.1038/s41746-025-01726-8 (PMC12122805; doi:10.1038/s41746-025-01726-8)

SUPPLEMENTARY MATERIALS

Supplementary Table 1: List of exclusions identified in phase 1, with reasons for exclusion.

Product	Manufacturer	Regulatory Approval & Class (EU, USA, Australia)	Reasons for Exclusion
CARA	Diagnos	FDA Class IIa	CARA is a picture archiving and communications system that performs enhancement and sharing of digital retinal images using a secure internet connection. CARA is not intended, nor marketed, to diagnose or grade any retinal abnormalities.<br><br>Excluded because it does not involve AI for ophthalmic imaging to inform clinical management.
CLARUS	Carl Zeiss Meditec, Inc	FDA Class II	The CLARUS 700 ophthalmic camera is indicated to capture, display, annotate and store images to aid in the diagnosis and monitoring of diseases and disorders occurring in the retina, ocular surface and visible adnexa. CLARUS 700 uses AI to automatically find the optic nerve head and accurately derive the patient's gaze rather than relying on internal fixation.<br><br>Excluded because it does not involve AI for ophthalmic imaging to help inform clinical management.
EyeLib	MIKAJAKI SA	CE Class I	Scanning platform that combines robotics and AI. Performs a 3D full body scan and face morphometry to obtain objective measurements of the eyes.<br><br>Excluded as EyeLib is considered AI in a medical device (AIiMD) rather than AI as a medical device (AIaMD) and is therefore out of scope for this review.
Image Quality Analyser	Vision Quest Biomedical, Inc.	FDA Class II	IQA is a software system intended for use in importing, displaying, analysing and managing images acquired with digital fundus cameras. An image quality output of "Adequate" or "Inadequate" is calculated automatically by IQA based on a set of pre-determined thresholds on three imaging artefacts (crescents, shadows, and blurriness). The image quality output can be used by the IQA user to determine whether a retinal image should be re-acquired or not, and IQA does not modify the retinal image.<br><br>Excluded because it does not inform clinical management.

IRIS Intelligent Retinal Imaging System	IRIS Intelligent Retinal Imaging Systems, LLC	FDA Class II	IRIS is a picture archiving and communications system and comprehensive web-based software system application intended for use in storing, managing, and displaying patient data, diagnostic data and images from computerised diagnostic instruments or systems. Original and colour amplified images can be viewed by trained healthcare professionals.<br><br>Excluded because it does not involve AI for ophthalmic imaging to inform clinical management.
RightEye Vision System	RightEye, LLC	FDA Class II	The RightEye Vision System is intended for recording, viewing, and analysing eye movements in support of identifying visual tracking impairment in human subjects, rather than for using ophthalmic imaging to help inform clinical management.
SCANLY home monitoring, powered by Notal OCT Analyser (NOA)	Notal Vision, Inc.	FDA Class II (De Novo Marketing Authorisation)	The Notal Vision Home OCT (NVHO) system, or SCANLY, is a device that consists of two elements: 1) Notal Home OCT device used by patients to self-image their eyes; at the end of each scanning session the data is transmitted via a secured wireless communication to the Notal Health Cloud. 2) Notal Health Cloud: cluster of servers and analysis units on which the Notal OCT Analyzer (NOA) analyses the scans received from the Notal Home OCT device. Processed data are presented on a dedicated interactive web-application, the Notal Home OCT Web Viewer.<br><br>Excluded as the product to be regulated is the NVHO system, of which NOA is a component, meaning that the NOA is considered AIiMD rather than AIaMD and is therefore out of scope for this review.
SOCT Software	Optopol Technology	CE Class IIa	SOCT utilises AI to de-noise the image quality, rather than informing clinical management.

Supplementary Table 2: PubMed search strategy and number of hits for each manufacturer, (date searched: 24 July 2024).

Note that some manufacturers may produce more than one AIaMD. In addition, the number of hits and included studies may exceed the total numbers reported in the results. This is because studies comparing two or more AIaMDs are counted separately for each AIaMD.

ID	Product	Manufacturer	PubMed Search Strategy	Hits	Included from PubMed Searches	Included from other sources
1	LumineticsCore (US) or IDx-DR (EU)	Digital Diagnostics Inc.	("LumineticsCore" OR "IDx-DR" OR "Digital Diagnostics") AND retin*	163	17	4
2	Eyeart	Eyenuk, Inc	"Eyeart" OR "Eyenuk"	49	21	1
3	Retmarker Screening or DAIRET (Italy)	METEDA S.r.l. (Manufacturer); Retmarker S.A. (developer, distributor)	"Retmarker" OR "DAIRET" OR "Meteda"	25	4	4
4	RetmarkerDR				1	0
5	SELENA+	eyRIS Pte. Ltd.	("eyRIS" OR "SELENA+") AND (retin* OR glaucoma* OR macula*)	50	2	2
6	Automated Disease Assessment (ARDA)	Verily Life Sciences	("Verily" OR "ARDA") AND retin*	55	4	5
7	Medios AI (or Medios DR)	Remidio Innovative Solutions Pvt. Ltd.	("Remidio" OR "Medios") AND retin*	19	6	2
8	OphtAI	Evolucare/ ADCIS (partnership)	"Evolucare" OR "ADCIS" OR "OphtAI"	25	1	2
9	RetCAD	Thirona Retina B.V.	("Thirona" OR "RetCAD") AND retin*	124	3	1
10	DeepDee	DeepDee	"DeepDee"	3	0	0
11	MONA DR	MONA.health	("MONA" OR "Vito") AND ("retin*" OR "glaucoma*")	196	1	0
12	MONA GLC				2	0
13	Retinalyze	RetinaLyze System A/S (Ltd.)	"Retinalyze"	5	3	4
14	AEYE-DS	AEYE Health	"AEYE" AND retin*	42	1	0
15	EyeCheckup	URAL TELEKOMÜNİKASYON SAN. TİC. A.Ş	"EyeCheckup" OR "URAL Telekom"	1	1	0
16	Reti-Eye Reti-CVD	Medi Whale Inc.	"Reti-Intelligence" OR "Reti-Eye" OR "Reti-CVD" OR "Medi Whale" OR "DrNoon"	24	5	0
17	ITOS Mass Screening	Voigtmann GmbH	("Voigtmann GmbH" OR "ITOS") AND retin*	0	0	0
18	Nexy A.I./ EyeWisdom MCS	Visionix, Luneau [Nexy]; Visionary Intelligence Ltd. [Nexy AI software]	("Nexy A.I." OR "EyeWisdom" OR "Visionix"	53	4	0

19	EyeWisdom DSS	Visionary Intelligence Ltd.	OR "Visionary Intelligence") AND retin*		3	0
20	RetInSight Fluid Monitor	RetInSight GmbH	"RetinSight" OR "Fluid monitor" OR "GA monitor"	45	2	4
21	RetInSight GA Monitor				1	0
22	RetinAI Layer Segmentation	Ikerian (formerly RetinAI Medical AG)	"RetinAI" OR "Ikerian"	42	0	1
23	RetinAI Fluid Segmentation				2	0
24	RetinAI Macula Biomarkers				2	0
25	iPredict	iHealthScreen Inc; Arif Systems	"iPredict" OR "iHealthScreen"	21	5	1
26	TeleMedC DR grader	TeleMedC PTE LTD	"TeleMedC" OR "RetinoScan"	1	2	2
27	Eyetelligence system (Assure Plus)	Eyetelligence Pty Ltd; Optain	"Eyetelligence" OR "Optain"	4	1	7
28	Eyetelligence system (Assure)				0	0
29	Diabetic Retinopathy Screening (DRISTi)	Artificial Learning Systems India Private Limited (Artelus)	"DRISTi" OR "Artelus"	14	0	0
30	VUNO Med - Fundus AI	VUNO	"VUNO"	96	2	0
31	BioAge	TokuEyes	"Toku Eyes"	7	1	0
32	CLAiR				1	0
33	iGradingM or AutoGrader	Medalytix Group Ltd -> Digital Healthcare -> EMIS Health	"iGradingM" OR "iGrading" OR "Autograder" OR "Medalytix"	4	4	3
34	Altris AI	Altris	"Altris"	0	0	0
35	Ophthal	mr-doc	“mr-doc”	6	0	0
36	UMI DR	ULMA Medical Technologies	“UMI DR” OR “ULMA”	90	0	0

Supplementary Table 3: Standardised data collection template emailed to manufacturers of AIaMDs identified in the review.

**Name of product**	
**Company**	
**Jurisdiction(s) for which approval given**<br>***(e.g. USA, Europe, UK, Aus etc)***	
**Class of device**<br>***(please specify for each regulator if more than one approval given)***	
**Intended use statement for the device**<br>***(please specify for each regulator if more than one approval given)***	
**Types of pre-market clinical evidence that supported its regulatory approval**<br>***(please X all that apply)***	**Study analysing performance on previously collected data [ ]**<br><br>**Study analysing performance on new, prospectively collected data [ ]**<br><br>**Study analysing performance as part of a 'silent trial'** ***(within the actual healthcare pathway but without acting on the findings)*** **[ ]**<br><br>**Study analysing performance as part of an interventional trial** ***(within the actual healthcare pathway and the output of the device influences patient care)*** **[ ]**
**Types of post-market clinical evidence that has been required by the regulator**<br>***(please specify including any ongoing data collection exercises or formal studies)***	
**Evidence for the device in the public domain (pre- or post-market)**<br>***(please provide links for any published study results; where these are not yet published please provide links to any clinical trial registrations or protocols)***	
**Evidence for the device that is not in the public domain (pre- or post-market)**<br>***(please provide any information that you are able to share; this may range from an outline description (e.g. type of clinical study and number of patients included) through to your full regulatory submission.)***	

Supplementary Figure 1: PRISMA flow diagram detailing study selection.

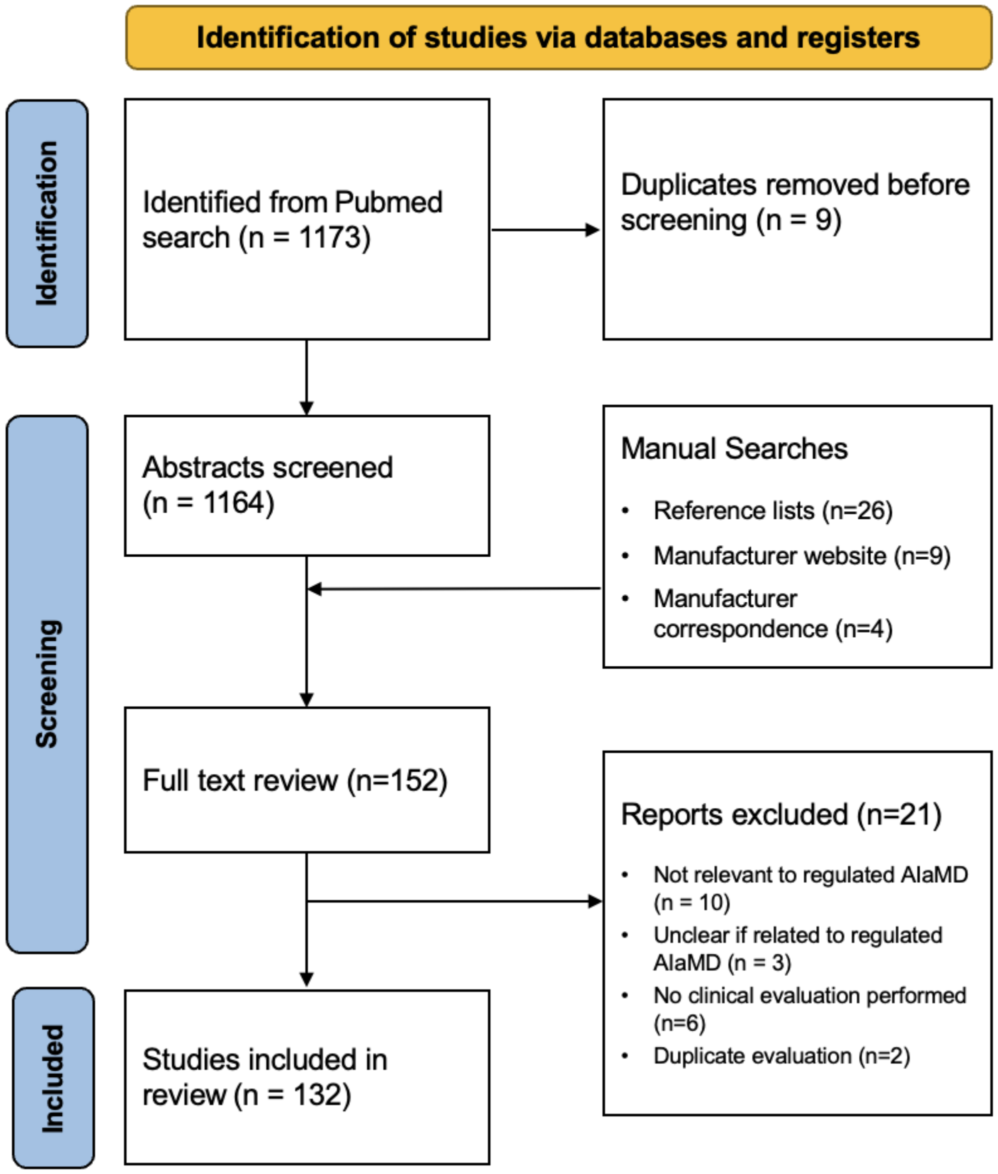

Supplement: Supplementary file 1 — Checklist [file 41746_2025_1726_MOESM1_ESM.pdf]
